# Prevalence of frailty and associated factors among Saudi community-dwelling older adults: a cross-sectional study

**DOI:** 10.1186/s12877-021-02142-9

**Published:** 2021-03-17

**Authors:** Bader A. Alqahtani, Aqeel M. Alenazi, Mohammed M. Alshehri, Ahmed M. Osailan, Saud F. Alsubaie, Mohammed A. Alqahtani

**Affiliations:** 1 Department of Health and Rehabilitation sciences, Prince Sattam Bin Abdulaziz University, Al-Kharj, 11942 Kingdom of Saudi Arabia; 2 Physical Therapy Department, Jazan University, Jazan, Kingdom of Saudi Arabia; 3 College of Medicine, Dar Al Uloom University, Riyadh, Saudi Arabia

**Keywords:** Frailty, Fried’s frailty, Pre-frail, Saudi Arabia, Older adults

## Abstract

**Background:**

Prevalence of frailty has been previously established in different Western countries; however, the prevalence and the burden of in the aging populations of Saudi Arabia has not been examined. Therefore, the aim of this study was to examine the prevalence of frailty, and associated factors among Saudi older population.

**Methods:**

The study included a total of 486 community-dwelling elderly adults aged 60 years and over living in the Riyadh area. This study took place from August 2019 to June 2020. The prevalence of frailty was determined using the Fried’s frailty phenotype. Association between sociodemographic features and clinical factors and frailty was estimated by Odds Ratio and confidence intervals (OR, IC 95%) using a multinomial logistic regression model.

**Results:**

The overall prevalence of pre-frailty and frailty were 47.3 and 21.4%, respectively. The following factors were associated with being frail: age (OR: 6.92; 95%CI 3.11–15.41); living alone (OR: 2.50; 95%CI: 1.12–5.59); had more chronic conditions (OR: 1.96; 95%CI: 1.16–3.30); and cognitive impairment (OR: 7.07; 95%CI: 3.92–12.74).

**Conclusions:**

The Compared with other populations, the prevalence of frailty and pre-frailty in the Riyadh region of Saudi Arabia was high. The implications of frailty in this population should be discussed in future study.

## Introduction

The aging population in Saudi Arabia will increase drastically over the next few decades. According to the United Nations estimates, the Saudi older population will increase from 5.6% in 2017 to 22.9% by 2050 [1]. With the increasing number of elderly people in Saudi Arabia, it places more burden to the healthcare system due to the high prevalence of comorbidities that needs close supervision and continuous care, such as diabetes, arthritis, cardiovascular diseases, and aging related conditions (e.g. frailty).

Aging has been associated with deterioration in different body systems which include musculoskeletal, cardiovascular, sensory, and cognition [2–4]. In addition, frailty has been related to aging, which can lead to increased risk of falling, greater vulnerability to adverse outcomes, increased functional limitations, and institutionalization [5–7]. Due to the current significant growth in elderly population in Saudi Arabia, the topic of frailty becomes more important now than ever [8].

Frailty is a clinical geriatric syndrome characterized by an excess vulnerability to adverse health outcomes [9, 10]. Frailty has become one of the serious public health issue in the geriatric population [11, 12]. A recent meta-analysis of studies from different populations has reported that the prevalence of frailty varied from 4% in Chinese older adults to 51% among older adults in Cuba [13].

Different measurements have been used to assess frailty [13]. Although, a gold standard measure has not been established yet, the Fried’s frailty phenotype is one of the most widely used measurements to assess frailty [14].The Fried’s frailty phenotype was put forth by Fried et al. using data from the Cardiovascular Health Study (CHS) [15]. Using this index, older adults are categorized as robust, pre-frail and frail based on five indicators include: unintentional weight loss, exhaustion, hand grip weakness, walking speed, and the level of physical activity. Investigators have demonstrated that the Fried’s frailty phenotype has been associated with falls, hospitalizations, disability and death [11].

Frailty is considered as a dynamic condition, with proper interventions frailty can be altered or improved (i.e. frail elderly can improve to become pre-frail or robust). However, without proper intervention, a deterioration for older adults may occur and become definitely more frail and susceptible to disability [16]. By taking the aforementioned facts into consideration, assessing the prevalence of frailty and its associated factors will help in building future plans to decrease the burden of frailty through implementing targeted interventions in early stages. Although numerous studies have been done on frailty in different countries, frailty status among Saudi older adults is unknown. Therefore, the main purpose of the current study was to investigate the prevalence of frailty, and examine the association between frailty and sociodemographic and associated clinical factors in Saudi older adults.

## Materials and methods

### Study design and participants

**Fig. 1.**
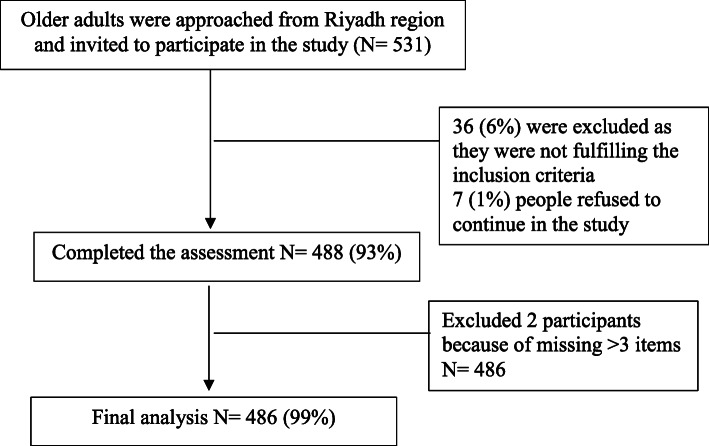
Flow chart of participants’ enrollment

This study was a community based cross-sectional study carried out in the Riyadh region specifically in Alkharj city, from August 2019 to June 2020. The estimated total population of the city in 2020 was about 425,300. With a great economic significance and up-to-date administration, Alkharj is one of the Kingdom’s main hubs. The city is rich in its valuable natural resources, has a broad geographical area and demographic diversity. Older adults aged 60 years and older who lives in Alkharj city were recruited to take part in the current study. Recruitment was accomplished mainly by advertising in media and local community and cooperation with local residential communities (i.e., social centers, residential district committees). All participants provided a written informed consent before enrolling in the present study. The study was approved by the ethical committee at Prince Sattam bin Abdulaziz University in accordance with the guidelines of the Helsinki Declaration for medical research involving human participants. Participants were included if they were aged ≥60 years. Participants were excluded if they were non-Saudi, had any acute disease or unstable medical condition that may affect the ability of answering the questionnaire proposed or complete the objective evaluation. More details about the participants enrollment are shown in Fig. 1.

### Study sample

The sample size for study participants was calculated using previously published formula for prevalence studies [17]. We used a prevalence of 28% from a previous pilot study that was done on small sample size [18], and the desired precision was set at 4%, with 95% confidence interval. Therefore, the final required sample was a total of 486 participants.

### Measurements

The Fried’s frailty phenotype was used to define and measure frailty in our study. The final score was based on the presence of 5 components: weight loss (measured by a self-report of unintentional weight loss of 10 pounds or more in the last year), exhaustion (defined by the participants responses as “I felt that everything I did was an effort” and “I could not get going” to questions adopted from the Center for Epidemiological Studies Depression (CES-D) scale), slow waling speed (measured by time spent to walk 15 ft (4.57 m), adjusted for gender and height), muscle weakness (measured by grip strength, using JAMAR PLUS+® digital hand dynamometer (Sam-mons Preston, Bolingbrook, IL, USA). Two trained physical therapists collected the strength data and the average of the peak force of the three measurements for the dominant hand was calculated by kilograms (kg). The calibration of hand dynamometer was tested periodically during the testing Grip strength data was stratified by gender and Body Mass Index (BMI), and low physical activity (assessed by using subject responses to the Minnesota Leisure Time Activities Questionnaire) [15]. Each component was assigned a score of 0 or 1. Participants were classified into 3 groups based on total score: 0 as robust, 1 to 2 as pre-frail, 3 or more as frail [15]. The Arabic version of the Mini-Mental State Examination (MMSE) was used to examine the cognitive function in the current sample [19]. The MMSE scores range from 0 to 30, with lower scores indicate poor cognitive function. A cutoff score of < 24 was used to identify participants with cognitive impairment [20]. Sociodemographic data include gender, age, marital status, living arrangements, education level were collected. Information on chronic conditions was collected using a self-report. Finally, the BMI was estimated as weight (kg) / height (m^2^). These measurements were obtained by trained physical therapists.

### Statistical analysis

Data was analyzed using statistical software Stata version 15.1 (Stata Corp, College Station, TX). For continuous sociodemographic variables the mean and standard deviation were reported, and percentages were used for categorical variables. Normality of the included variables was assessed using Kolmogorov-Smirnov test and the data were normally distributed. One-way analysis of variance was used to compare baseline characteristics between frailty, pre-frailty and robust groups for continuous variables and chi-square test was used for categorical variables. Variables with *p*-value < 0.10 on the univariaable analysis were then selected for multinomial regression analysis. A multinomial logistic regression model was constructed to examine the association between sociodemographic characteristics and clinical factors and frailty groups. The robust group was used as the reference group. Model goodness of fit was checked using the deviance statistic and the pseudo R (Nagelkerke) and unadjusted odds ratio (OR) with the 95% confidence intervals were reported. The level of statistical significance was set at α ≤ 0.05.

## Results

A total of 486 participants were recruited in the current study. Table 1 shows the basic demographic and clinical characteristics of the participants. The average age of our sample was 71 years (range 60–89 years). Sixty-five percent (317/486) of the participants were male. The prevalence of frailty and its components are presented in Table 2. A total of 21.4% of participants were frail (females, 22.7%), 47.3% were pre-frail (females, 51.5%), and 31.2% were robust (females, 32.2%). Females reported higher prevalence of exhaustion (35.3%), weakness (36.2%), and low physical activity (32.8%) as shown in Table 2.

**Table 1:** Sociodemographic and clinical characteristics of the study sample according to frailty status

Variable	Total sample ***N*** = 486 (%)	Frailty Status, n (%)			***p***
		Robust	Pre-frail	Frail	
**Age groups**					
**60–69**	**218 (44.8)**	**79 (36.2)**	**117 (53.6)**	**22 (10.1)**	**< 0.001**
**70–79**	**198 (40.7)**	**59 (29.8)**	**84 (42.4)**	**55 (27.8)**	
**≥ 80**	**70 (14.4)**	**14 (20.0)**	**29 (41.4)**	**27 (38.6)**	
**Gender**					
**Male**	**317 (65.2)**	**102 (32.2)**	**143 (45.1)**	**72 (22.7)**	**0.384**
**Female**	**169 (34.7)**	**50 (29.6)**	**87 (51.5)**	**32 (18.9)**	
**Education**					
**No formal education**	**286 (61.3)**	**93 (32.5)**	**128 (44.7)**	**65 (22.7)**	**< 0.001**
**Primary school**	**119 (24.4)**	**39 (36.8)**	**58 (54.7)**	**9 (7.5)**	
**Middle school or more**	**81 (16.6)**	**14 (17.3)**	**37 (45.7)**	**30 (37.0)**	
**Marital status**					
**Married**	**298 (61.3)**	**107 (35.9)**	**144 (48.3)**	**47 (15.8)**	**< 0.001**
**Single/widowed/divorced**	**188 (38.7)**	**45 (23.9)**	**86 (45.7)**	**57 (30.3)**	
**Living arrangement**					
Living with others	**425 (87.4)**	**141 (33.2)**	**197 (46.3)**	**87 (20.5)**	**0.051**
Living alone	**61 (12.6)**	**11 (18.0)**	**33 (54.1)**	**17 (27.9)**	
**Number of chronic conditions**					
**None**	**115 (23.6)**	**46 (40.0)**	**59 (51.3)**	**10 (8.7)**	**< 0.001**
**1**	**110 (22.6)**	**26 (23.6)**	**66 (60.0)**	**18 (16.4)**	
**2 or more**	**261 (53.7)**	**80 (30.6)**	**105 (40.2)**	**76 (29.1)**	
**Grip strength, mean (SD)**	**486**	**19.3 (8.8)**	**16.9 (9.8)**	**6.66 (5.8)**	**< 0.001**
**BMI (Kg/m**^**2**^**), mean (SD)**	**486**	**27.5 (4.8)**	**26.0 (4.9)**	**23.7 (4.8)**	**< 0.001**

**Table 2:** **Prevalence of frailty status and its components**

	Male (***n*** = 317) % (CI)	Female (***n*** = 169) % (CI)	Total (***n*** = 486) % (CI)
**Frailty status**			
**Frail**	**22.7 (18.4–27.6)**	**18.9 (13.7–25.5)**	**21.4 (17.9–25.2)**
**Pre-frail**	**45.1 (39.6–50.6)**	**51.5 (43.9–58.9)**	**47.3 (42.9–51.7)**
**Robust**	**32.2 (27.2–37.5)**	**29.5 (23.1–36.9)**	**31.2 (27.2–35.5)**
**Frailty components**			
**Weight loss**	**11.4 (8.3–15.40)**	**11.8 (7.7–17.6)**	**11.5 (8.9–14.6)**
**Exhaustion**	**35.3 (30.2–40.8)**	**33.1 (26.4–40.6)**	**34.5 (30.4–38.9)**
**Slow gait speed**	**22.7 (18.4–27.6)**	**25.4 (19.4–32.5)**	**23.6 (20.1–27.6)**
**Weakness**	**36.2 (31.1–41.2)**	**23.1 (17.3–30.1)**	**31.6 (27.6–35.9)**
**Low physical activity**	**32.8 (27.8–38.2)**	**27.8 (21.5–35.1)**	**31.1 (27.1–35.3)**

Table 3 show the results of association between the participants’ sociodemographic characteristics and frailty status in adjusted and unadjusted multinomial regression models, with robust participants as a reference group. Significant associations were found for all variables that were included in the model except for gender. Frail participants were older (OR: 6.92; 95%CI 3.11–15.41), were more likely to live alone (OR: 2.50; 95%CI: 1.12–5.59), had more chronic conditions (OR: 1.96; 95%CI: 1.16–3.30), and had lower cognitive function (OR: 7.07; 95%CI: 3.92–12.74) than those who were robust. In the unadjusted model, live alone (OR: 2.15; 95%CI: 1.05–4.39), had more chronic conditions (OR: 0.59; 95%CI: 0.37–0.95), and had lower cognitive function (OR: 2.10; 95%CI: 1.21–3.51) were associated with pre-frailty. Associations were remained significant after adjusting for gender and age.

**Table 3:** Association between sociodemographic characteristics and frailty status in adjusted and unadjusted multinomial regression models

Variable	Unadjusted model		Adjusted model	
	Robust vs pre-frail	Robust vs frail	Robust vs pre-frail	Robust vs frail
	RRR (95% CI)	RRR (95% CI)	RRR (95% CI)	RRR (95% CI)
**Age groups**				
**60–69**	**Ref.**	**Ref.**	**Ref.**	**Ref.**
**70–79**	0.96 (0.62–1.49)	**3.35 (1.83–6.10)**	0.95 (0.61–1.48)	**3.36 (1.83–6.10)**
**≥ 80**	1.40 (0.69–2.82)	**6.92 (3.11–15.41)**	1.37 (0.68–2.70)	**7.04 (3.15–15.70)**
**Gender**				
**Men**	**Ref.**	**Ref.**	**Ref.**	**Ref.**
**Women**	0.81 (0.52–1.24)	1.10 (0.64–1.88)	0.81 (0.53–1.25)	1.21 (0.69–2.12)
**Living alone**				
**No**	**Ref.**	**Ref.**	**Ref.**	**Ref.**
**Yes**	**2.15 (1.05–4.39)**	**2.50 (1.12–5.59)**	**2.21 (1.07–4.58)**	**2.01 (1.06–4.62)**
**3 or more chronic conditions**				
**No**	**Ref.**	**Ref.**	**Ref.**	**Ref.**
**Yes**	**0.59 (0.37–0.95)**	**1.96 (1.16–3.30)**	**0.61 (0.37–0.98)**	**2.27 (1.30–3.95)**
**Cognitive status**				
**Normal**	**Ref.**	**Ref.**	**Ref.**	**Ref.**
**Impaired**	**2.10 (1.21–3.51)**	**7.07 (3.92–12.74)**	**2.21 (1.26–3.85)**	**5.45 (2.91–10.21)**

## Discussion

This study examined the prevalence of frailty in Saudi older adults and associated factors. Our findings showed that the overall prevalence of pre-frailty and frailty were 47.3 and 21.4%, respectively. The current study also identified the associated factors for frailty including older age, living alone, having 3 or more comorbidities, and impaired cognitive status. Although several studies have investigated the prevalence of frailty in different countries, there is limited information of frailty in Saudi Arabia. It is imperative to understand the associated health changes amongst Saudi population since the incidence of chronic health issues in Saudi Arabia differ to other countries in middle east. To our knowledge, this study provided first findings about frailty status using reliable and valid measurement and analyzed via structured methods among Saudi population.

The results of our study indicated that the prevalence of frailty in people aged 60 years or older in Saudi Arabia is 21.4%. This prevalence is slightly higher than most of other studies that found the prevalence of frailty in several different countries between 4 and 16% [21–24]. On the other hand, a number of previous studies have shown that the prevalence of frailty can be even higher. One study conducted in Cuba and found that the prevalence rate reached about 51% of the elderly [13].

The differences in prevalence reported by previous studies may be driven by methodological differences such as the age of participants as well as the population of the study; Studies have found that the prevalence of frailty is higher in those who reside in medical care centers than in the general community [21–25]. Studies also differed in the method of identifying of frailty; one approach of finding the prevalence of frailty is to follow Fried’s frailty criteria [15], which is used in most studies, including our study. In contrast, few studies have adopted the cumulative deficits scores criteria which depends on the accumulative number of deficits in different organs of the body as an indicator of frailty [26].

The prevalence of pre-frailty was found to be 47.3%, which is consistent with the results of other studies [23, 27–30]. However, the prevalence of pre-frailty is relatively high and should be considered as an indication of future frailty, and prevention programs should be established early.

The results of our study were consistent with results of several studies that have reported that the prevalence of frailty is strongly associated with cognitive impairment [23, 31–34]. Further, a number of studies have found that the presence of frailty is a significant predictor of future cognitive impairment [33, 34]. Similar to many studies, our study found that the prevalence of frailty is associated with having three or more chronic conditions [23, 31–34]. Our study also found an association between frailty and living alone, and this relationship is not clear because a few numbers of studies have looked at this relationship. However, living alone in Saudi Arabia may not be a common factor in older adults due to cultural differences when compared to other countries. Therefore, we selected this explanatory variable to examine the association between living alone and frailty status, and it was a significant association. This will add to the literature about Saudi Arabia for further research to understand the reasons behind this association such as diet, exercise, psychological, and other factors.

The prevalence of frailty increased sharply with increasing age as expected and reported in several studies [23, 25, 28, 31, 32, 35]. On the contrary, there was no difference between male and female in the prevalence of frailty, while several studies have indicated that the prevalence of frailty is higher in female [27, 31, 32, 35].

According to the General Authority for Statistics in Saudi Arabia, it was noticed that there is increase in the ratio of Saudi citizens who are at least 65 years old [36]. Therefore, as the population of older people in Saudi Arabia increase, prevalence of frailty will undoubtedly increase. This may result in a significant increase in hospital admissions and prolonged length of stay at the hospital.

National Health Service in the United Kingdom reported high increase in older people (75 years old and above) hospitalization from 2,308,480 in 2000 up to 3,837,990 in 2010 [37, 38], were older people with frailty represented a large proportion of these admissions. Furthermore, Frail people have high susceptibility for hospital re-admission in a short period of time [39]. It was also found that the hospitalization period was longer [40], and mortality rate was higher [41], in frail people than non-frail people.

Given the dearth of the mentioned evidence and the prevalence of frailty in Saudi Arabia reported in the present study, it is crucial to develop strategies and intervention to alter or reduce frailty. Interventions should be aimed to reduce frailty in order to cut the costs of prolonged hospitalization, hospital re-admission, and other associated comorbidities. Using Fried’s frailty phenotype score as a measure of frailty status in clinics is one the first steps that can allow early identification of frailty status and the associated factors as well, which eventually, lead to better planning of future strategies and intervention at a population level either to prevent, reduce or to invert frailty.

Several limitations in this study should be recognized. Although Riyadh region is the largest diverse community in Saudi Arabia, there is a need to assess the prevalence of frailty status in all regions of Saudi Arabia to allow generalizability. It has been reported that several medications correlated with frailty status such as hypnotics, analgesics, and laxatives [42]. In addition, some mediations are prescribed to decrease the frailty symptoms [43]. Thus, there is a need to control for medications to strengthen the results of future researches. The cross-sectional design of this study is another limitation that limit causality relationship. Although this design is considered a limitation in population-based research, our study identified the associated factors with frailty status, and this can be achieved by cross-sectional design. Since there is a limited evidence about frailty in Saudi Arabia, we need to examine the prevalence first and look at the associated factors with this condition. Then, when we have data and evidence, we can identify predictors for frailty in this population using longitudinal design. Other variables should be considered in future studies such as falls and hospitalization, depressive symptoms, functional capacity, and, health perception. Number of outcomes in this study were based on self-reported outcomes which might not be sensitive enough to present the accurate associations of frailty status in Saudi population. Some of domains in the frailty status might be overestimated by self-reported measures [44]. Therefore, we recommend using objective measures to accurately represent domains related frailty status in future research. Finally, depression and anxiety symptoms are highly associated with frailty status in general population [45]. We did not control for psychological symptoms which is needed to avoid extraneous effect on the prevalence and the relationship of frailty with demographics and clinical factors in Saudi population.

## Conclusions

This study underlines the high level of prevalence of frailty and prefrailty among Saudi older adults. Both frailty and pre-frailty were associated with living alone, multiple comorbidities, and impaired cognitive function As the prevalence of frailty is high, we recommend raising awareness about this condition and its effect on older adults, and health practitioners. Assessment for fraility should be addressed in primary care settings. Future research should examine frailty across different regions in Saudi Arabia.

## Data Availability

The datasets used and/or analysed during the current study are available from the corresponding author on reasonable request.

## Abbreviations

CHS: Cardiovascular Health Study
CES-D: Epidemiological Studies Depression
BMI: Body Mass Index
MMSE: Mini-Mental State Examination
ANOVA: Analysis of variance
OR: Odds ratios
